# Personalized monitoring of ambulatory function with a smartphone 2-minute walk test in multiple sclerosis

**DOI:** 10.1177/13524585231152433

**Published:** 2023-02-08

**Authors:** Ka-Hoo Lam, Ioan Gabriel Bucur, Pim van Oirschot, Frank de Graaf, Eva Strijbis, Bernard Uitdehaag, Tom Heskes, Joep Killestein, Vincent de Groot

**Affiliations:** Department of Neurology, Amsterdam University Medical Centers, Universiteit Amsterdam, Amsterdam, The Netherlands/MS Center Amsterdam, Amsterdam, The Netherlands/Amsterdam Neuroscience, Amsterdam, The Netherlands; Institute for Computing and Information Sciences, Radboud University, Nijmegen, The Netherlands; MS Sherpa BV, Nijmegen, The Netherlands; Orikami Digital Health Products, Nijmegen, The Netherlands; Department of Neurology, Amsterdam University Medical Centers, Universiteit Amsterdam, Amsterdam, The Netherlands/MS Center Amsterdam, Amsterdam, The Netherlands/Amsterdam Neuroscience, Amsterdam, The Netherlands; Department of Neurology, Amsterdam University Medical Centers, Universiteit Amsterdam, Amsterdam, The Netherlands/MS Center Amsterdam, Amsterdam, The Netherlands/Amsterdam Neuroscience, Amsterdam, The Netherlands; Institute for Computing and Information Sciences, Radboud University, Nijmegen, The Netherlands; Department of Neurology, Amsterdam University Medical Centers, Universiteit Amsterdam, Amsterdam, The Netherlands/MS Center Amsterdam, Amsterdam, The Netherlands/Amsterdam Neuroscience, Amsterdam, The Netherlands; MS Center Amsterdam, Amsterdam, The Netherlands/Amsterdam Neuroscience, Amsterdam, The Netherlands/Department of Rehabilitation Medicine, Amsterdam University Medical Centers, Vrije Universiteit Amsterdam, Amsterdam, The Netherlands

**Keywords:** Multiple sclerosis, ambulatory function, outpatient monitoring, smartphone, digital technology, patient-specific modeling

## Abstract

**Background:**

Remote smartphone-based 2-minute walking tests (s2MWTs) allow frequent and potentially sensitive measurements of ambulatory function.

**Objective:**

To investigate the s2MWT on assessment of, and responsiveness to change in ambulatory function in MS.

**Methods:**

One hundred two multiple sclerosis (MS) patients and 24 healthy controls (HCs) performed weekly s2MWTs on self-owned smartphones for 12 and 3 months, respectively. The timed 25-foot walk test (T25FW) and Expanded Disability Status Scale (EDSS) were assessed at 3-month intervals. Anchor-based (using T25FW and EDSS) and distribution-based (curve fitting) methods were used to assess responsiveness of the s2MWT. A local linear trend model was used to fit weekly s2MWT scores of individual patients.

**Results:**

A total of 4811 and 355 s2MWT scores were obtained in patients (*n* = 94) and HC (*n* = 22), respectively. s2MWT demonstrated large variability (65.6 m) compared to the average score (129.5 m), and was inadequately responsive to anchor-based change in clinical outcomes. Curve fitting separated the trend from noise in high temporal resolution individual-level data, and statistically reliable changes were detected in 45% of patients.

**Conclusions:**

In group-level analyses, clinically relevant change was insufficiently detected due to large variability with sporadic measurements. Individual-level curve fitting reduced the variability in s2MWT, enabling the detection of statistically reliable change in ambulatory function.

## Introduction

Ambulatory dysfunction interferes with daily living in the majority of patients with multiple sclerosis (MS) at some point during the disease.^1^ Ambulation is considered among the most important functional spheres,^2^ and dysfunction contributes substantially to patient burden.^3^ Correspondingly, walking impairment is important as a marker of MS severity and progression. Accumulation of disability starts early in MS due to neurodegeneration or relapse activity,^4^ yet the time of onset and the extent is highly variable between as well as within patients.^5^ Therefore, timely and targeted disease management is crucial and also highly patient-specific, and would gain from accurate and sensitive assessment on the individual patient level.^6^

The Expanded Disability Status Scale (EDSS) is most universally used to quantify MS severity in clinical practice and trials.^7^ The EDSS suffers, however, from high inter- and intra-rater variability, and is poorly responsive to clinically relevant changes.^8–10^ For instance, despite emphasis on ambulation in patients with moderate disability, the EDSS does not reflect change in ambulation if a patient is able to walk 500 m or more.^9^ Therefore, clinical measures specific for ambulation, such as the timed 25-foot walk test (T25FW) and the 12-item MS Walking Scale, are intended to capture ambulation more sensitively.^11,12^ These measures have been extensively validated with established cut-offs for clinically relevant change and are often used as outcomes in clinical trials.^13,14^ Despite this, clinical measures do not always reflect real-life functioning and agreement on the individual level is often lacking, making clinically important change difficult to distinguish from measurement variability.^15,16^ Therefore, in clinical practice, where statistical power cannot be drawn from large samples or group analyses, there is no consensus on the use of these outcomes to monitor individual patients longitudinally for decision-making purposes in MS.^17^

Increasingly, technological solutions are being developed and studied to offer potential advantages compared to clinical measurement tools.^18^ Digital devices may remotely capture the patient’s function in the real-life environment, during actual tasks of daily living, and at a much higher frequency. This may allow sufficient sensitivity for individual-based monitoring. Recent studies using smartphones to assess ambulatory function demonstrated reliability and validity.^19–26^ These studies reported cross-sectional analyses and mostly used a preconfigured smartphone provided for the study for validation purposes. In a previous study, a smartphone-based 2-minute walk test (s2MWT) was validated on participants’ self-owned devices in a 4-week follow-up.^27^ To advance this validation work toward clinical practice, the s2MWT needs to be assessed on relevant clinimetric properties and analyzed in the longitudinal setting.

### Objective

To investigate an s2MWT on the assessment of, and (anchor- and distribution-based) responsiveness to ambulatory function, to monitor function in MS.

## Methods

### Participants and study design

The study comprised a cohort evaluating the smartphone for assessing MS at the Amsterdam University Medical Centers, location VU University medical center. The study design has been reported previously in a baseline interim-analysis.^28^ Briefly, MS patients and healthy controls (HCs) were consecutively included from August 2018 until a sample size of approximately 100 patients and 25 HCs was reached in December 2019. Participants used the MS sherpa^®^ app to self-administer tests in their home environment and underwent three monthly clinical visits. Follow-up duration was 12 months for patients (visits at M_0_, M_3_, M_6_, M_9_, and M_12_) and 3 months (visits at M_0_ and M_3_) for HC. Eligibility criteria at baseline screening were: age between 18 and 65 years, use of a smartphone (Android version ⩾ 5.0 or iOS version ⩾ 10), no visual or upper limb deficits affecting regular smartphone use, no mood or sleep disorder impacting daily living, and, additionally for patients, a definite MS diagnosis and an EDSS score < 7.5. The study was approved by the medical ethical reviewing committee. Written informed consent was received from all participants.

### Study outcomes

During each clinical visit, the overall severity of disability due to MS and ambulatory function were assessed with the EDSS and T25FW, respectively. The EDSS is a measure based on the neurological examination ranging from 0 (normal exam) to 10 (death due to MS).^29^ Clinically relevant change on the EDSS was defined as ⩾1.5-point change from EDSS = 0, ⩾1-point change from EDSS = 1.0–5.5, and ⩾0.5-point change from EDSS ⩾ 6.0.^29^ With the T25FW, the average time needed to walk a distance of 25 feet (7.6 m) over two trials was scored.^11^ A ⩾20% change in T25FW was considered clinically relevant and used as anchor in the responsiveness analysis.^30^

### Smartphone walking tests

Two-minute walking tests (2MWTs) were performed with the MS sherpa app (Sherpa BV, https://www.mssherpa.nl/en/). The MS sherpa s2MWT was scheduled twice every 3 days during the initial 4 weeks and thereafter once a week for the remainder of the study. Participants were notified with push-notifications whenever an s2MWT was scheduled. The s2MWT measured the distance walked during 2 minutes based on location data of the GPS. The data were then sent to the database and an algorithm calculated the walked distance. App instructions for the participants were: walk as fast (and safely) as possible without running or jogging, preferably in a straight line, and the same route during each test. The smartphone vibrates and rings to indicate the start and end of the test.

### Statistical analysis

Statistical analyses were performed with IBM SPSS Statistics 28 and curve fitting with Python 3.9.5 (statsmodels package). Categorical data were summarized with frequencies and percentages, and analyzed with Fisher’s exact tests. Numerical data were summarized using the mean ± *SD* (or median and interquartile range (IQR) if not normally distributed), and analyzed with *t*-tests (or Mann–Whitney *U* tests). *P*-values below 0.05 were considered statistically significant.

#### Assessment of ambulatory function

The s2MWT was investigated for adherence, reproducibility, and validity for the assessment of ambulatory function. Adherence was calculated as the number of completed tests as percentage of scheduled tests in the initial 4-week period, between week 5 and M_3_, M_3_–M_6_, M_6_–M_9_, and M_9_–M_12_. To determine reproducibility, two s2MWT scores (test and retest) were used within a 2-week interval, where no real change can be assumed. s2MWT scores within the initial 4-week period were omitted in this analysis due to the higher scheduled frequency compared to the subsequent weekly schedule. Reproducibility parameters were: reliability (intra-class correlation coefficient (ICC); single measure, one-way random model on absolute agreement) and agreement (standard error of measurement, $SEM=SD\times \sqrt{1-ICC}$ , and smallest detectable change, $SDC=1.96\times \sqrt{2}\times SEM$ ).^31^ Validity was assessed in terms of construct and concurrent validity. For construct validity, median s2MWT scores were compared (Mann–Whitney *U* test) between MS patients stratified as (1) “low disability,” (2) “high disability,” and (3) HC. Stratification into low or high disability was done using the median split in T25FW and EDSS. For concurrent validity, the degree to which the s2MWT correlated (Spearman’s correlation coefficient *ρ*) with the EDSS and T25FW was determined. s2MWT scores within 7 days of the clinical visits were averaged. Correlation coefficient sizes of < 0.3, 0.3–0.6, and > 0.6 were considered low, moderate, and strong, respectively.^31^

#### Responsiveness to ambulatory function

To assess the s2MWT for monitoring ambulatory function, responsiveness was analyzed on the group and individual levels. For the group-level analysis, an anchor-based method was used to assess the sensitivity of changes in s2MWT to relevant changes in T25FW (⩾ 20% change was anchored as relevant)^13^ and EDSS (changes of ⩾ 1.5, ⩾ 1.0, or ⩾ 0.5 points were relevant if reference EDSS was 0, between 1.0 and 5.5, or ⩾ 6.0, respectively).^29^ At each clinical visit, s2MWT scores within 7 days were averaged, from which changes were calculated that correspond to the 3-month intervals of clinical outcomes. To account for correlated observations (multiple 3-month intervals) within patients, weighted averages were calculated and used, similar to repeated measures correlation analyses.^32^ Responsiveness was then quantified from the area under the receiver-operating characteristics curve (area under the curve (AUC)) where the true-positive rate ( sensitivity ) was plotted against the false-positive rate ( 1−specificity ). AUC values ⩾ 0.70 were indicative of adequate responsiveness.^31^ The most optimal cut-point value of change in s2MWT (the highest Youden’s *J*-statistic, J=sensitivity+specificity−1 ),^33^ was determined as the minimal clinically important difference (MCID) and compared to the smallest detectable change (SDC). If the MCID value exceeds the SDC value, important change can be reliably distinguished from measurement error.^31^

For responsiveness of the s2MWT on the individual level, a local linear trend model (LLTM) was used to fit an estimated curve through all data points of individual patients. This partly addresses measurement variability of frequent sampling due to factors unrelated to ambulatory function as noise, while allowing visualization of the estimated s2MWT level and its 95% confidence interval (CI) bands over time. This curve fitting method was reported earlier for a smartphone-based cognition test.^34^ We defined statistically reliable change in s2MWT level when there is no overlap between 95% CI bands. A detailed description of the model and the algorithm for statistically relevant change can be found in the Supplementary material.

## Results

**Figure 1.**
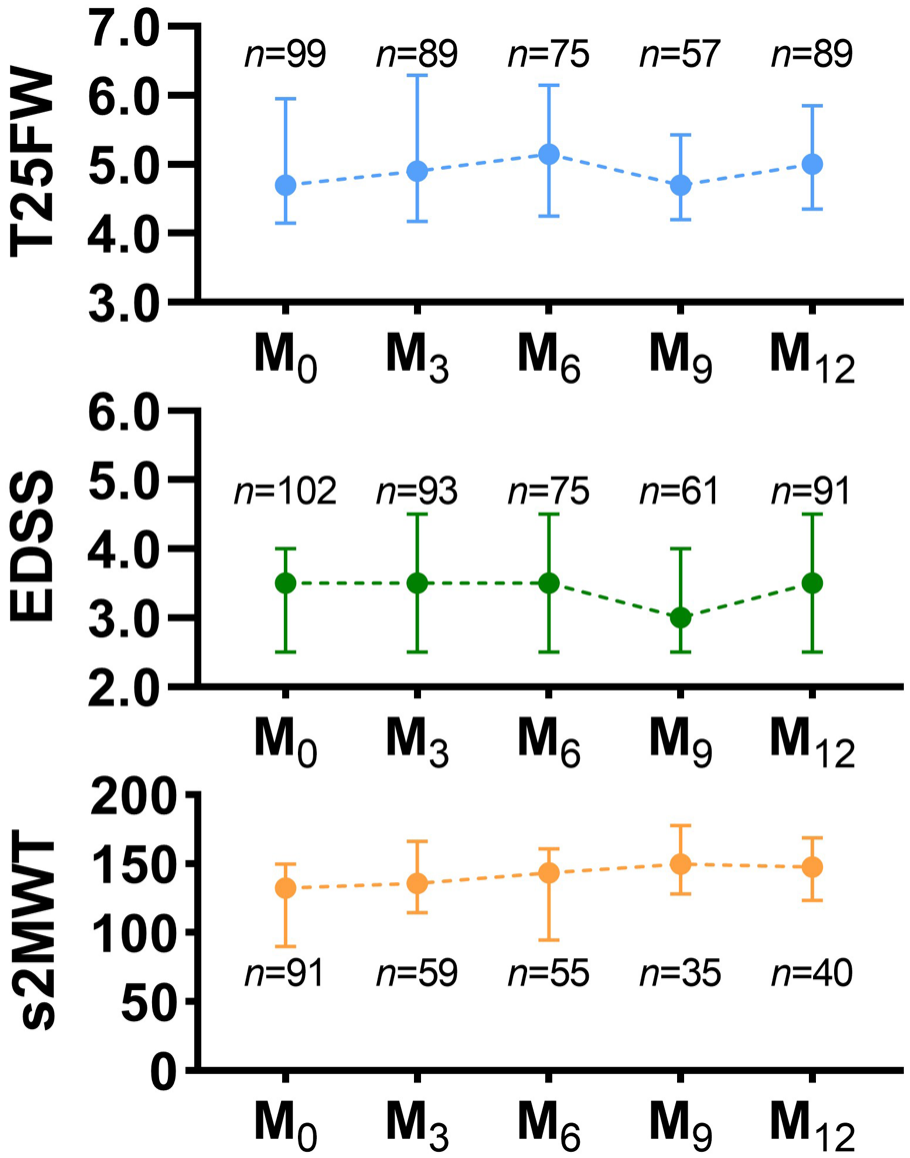
Line graphs of clinical outcomes and 2-week averaged s2MWT at each clinical visit from baseline (M_0_) to 12-month follow-up (M_12_). T25FW in seconds, EDSS score, and s2MWT in meters. Symbols depict the median value and error bars represent the interquartile range. Abbreviations: T25FW: timed 25-foot walk test; EDSS: Expanded Disability Status Scale; s2MWT: smartphone-based 2-minute walk test.

In total, 102 MS patients and 24 HCs were included in the study. Six patients and one HC dropped out at M_3_, one patient each at M_6_ and M_9_, and three patients at M_12_. Of the 102 MS patients and 24 HCs, smartphone walking tests were obtained in 94 patients and 22 HCs. The median (IQR) follow-up duration of the 94 patients was 392 (363–427) days, and 91 (80–96) days for the 22 HCs. The baseline demographical and clinical characteristics are summarized in Table 1. The scores of the study outcomes and s2MWT at each clinical visit are shown in Figure 1.

**Table 1.** Baseline demographical, clinical, and smartphone characteristics.

	MS patients (*n* = 94)	HC (*n* = 22)	*p*-value
Age, years, mean (*SD*)	46.5 (10.6)	43.9 (14.9)	0.450^a^
Sex, *n* (%)			
Female	68 (72.3)	12 (54.5)	0.104^b^
Male	26 (27.7)	10 (45.5)	
MS type, *n* (%)			
PPMS	11 (11.7)		n/a
SPMS	25 (26.6)		
RRMS	58 (61.7)		
Disease duration, years, median (IQR)	5.6 (2.9–13.1)		n/a
EDSS, median (range)	3.5 (1.5–7.0)		n/a

Abbreviations: MS, multiple sclerosis; HC: healthy control; IQR: interquartile range; PPMS: primary progressive multiple sclerosis; SPMS: secondary progressive multiple sclerosis; RRMS: relapsing–remitting multiple sclerosis; EDSS: Expanded Disability Status Scale.

a Independent *t*-test.

b Chi-squared test.

### Assessment of ambulatory function

**Figure 2.**
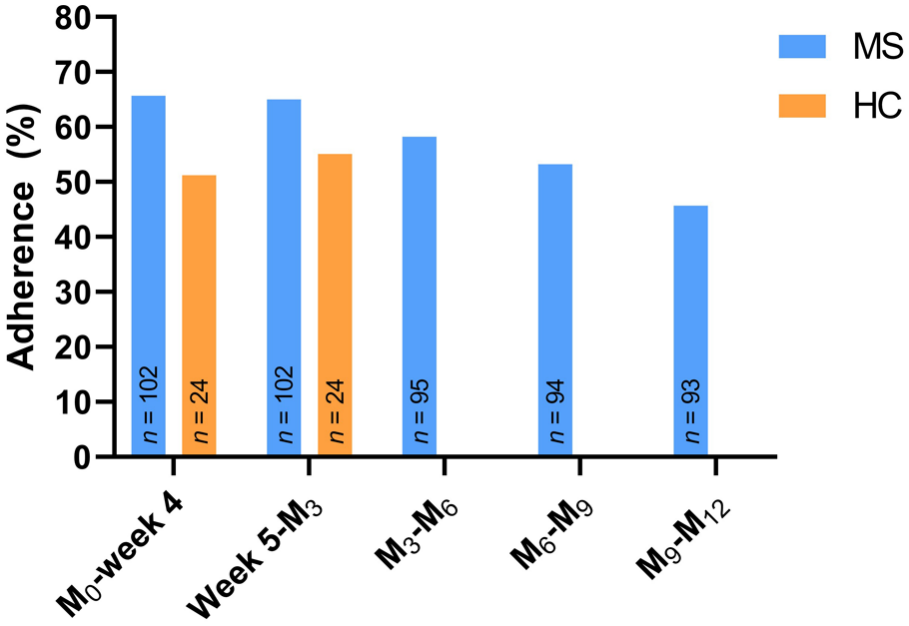
Adherence rates to the scheduled s2MWT throughout the study period. Abbreviations: MS: multiple sclerosis; HC: healthy control.

MS patients completed 6144 smartphone walking tests and HC completed 554 tests. The adherence rates to the s2MWT are shown in Figure 2. Of the 6144 (554 in HC) tests, 21.7% (35.9% in HC) were removed: 11.6% (HC 16.4%) insufficient GPS signal quality or inaccurate test duration; 8.6% (HC 19.3%) unreliable walking distance due to an older app version; 1.4% (HC 0.2%) not uploaded from the smartphone. The remaining 78.3% and 64.1% tests were obtained in 94 patients and 22 HCs, respectively, and included in the analyses. The median (IQR) number of tests per participant was 35 (16–60) in patients and 11 (3–16) in HCs.

For the test–retest analyses, 74 of the 94 patients had an s2MWT “test” (median of 29.0 (IQR = 29.0–36.0) days from baseline) and “retest” (median of 36.0 (IQR = 36.0–47.0) days from baseline) score within a 2-week interval. The following reproducibility parameters were obtained: ICC = 0.764 (95% CI [0.651, 0.845]), SEM = 23.63 m, and SDC = 65.50 m. For construct validity, 2-week averaged s2MWT scores were obtained in 75 MS patients and 16 HC at M_0_. Significant differences in s2MWT scores were found between: “low T25FW” versus “high T25FW,” “low EDSS” versus “high EDSS,” and HC versus “high EDSS” groups (see also Supplemental Figure S1). For concurrent validity, s2MWT scores correlated moderately to strongly with T25FW (*ρ* values between −0.43 and −0.61) and EDSS (*ρ* values between −0.44 and −0.64) (see also Supplemental Table S1).

### Responsiveness to ambulatory function

Considering 3-month intervals in the 94 MS patients, there were 15 (of 131) and 18 (of 127) intervals with clinically relevant change on the T25FW and EDSS, respectively. The s2MWT was insufficiently responsive to detect these changes in T25FW (AUC = 0.592, 95% CI [0.431, 0.753], MCID = 31.9 m) or EDSS (AUC = 0.482. 95% CI [0.333, 0.632], MCID = 29.7 m) as AUC values did not exceed 0.70. The MCID values were also lower than the SDC (65.5 m) found in the reproducibility analysis, and therefore, cannot be reliably distinguished from measurement error.

**Figure 3.**
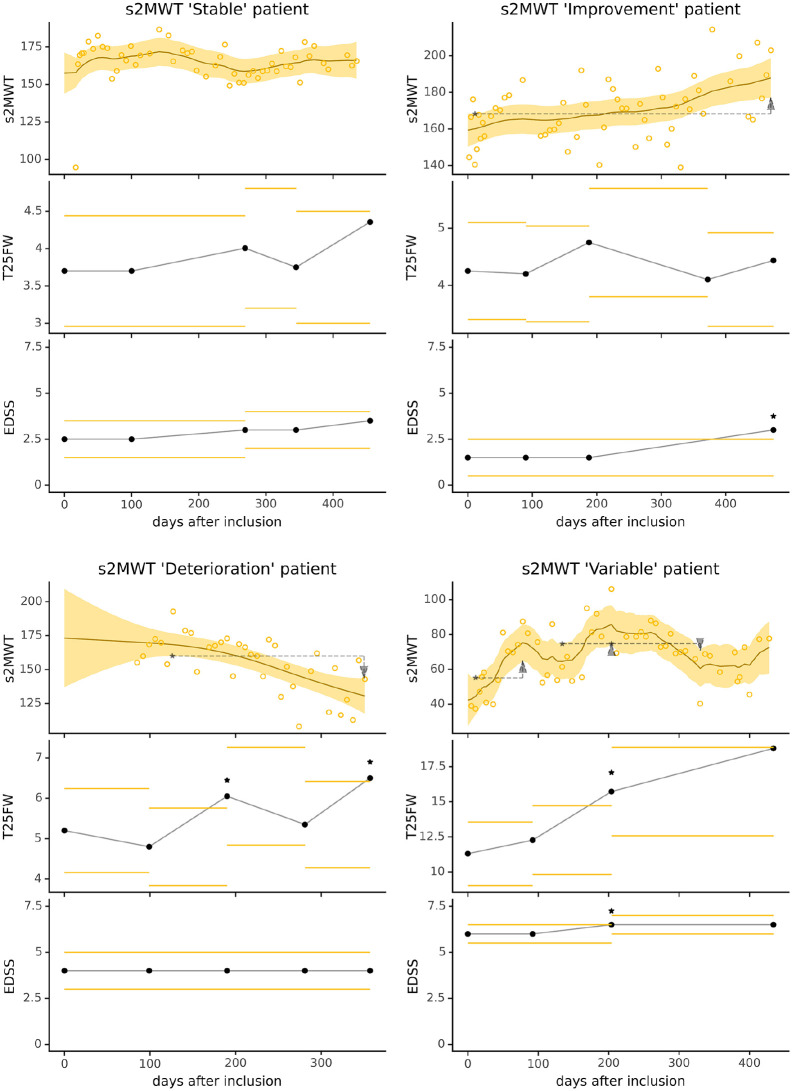
Curve fitting examples showing each of the four most distinct trajectories in smartphone walking function. Each graph encompasses all s2MWT data of a single patient. The upper panels show the s2MWT scores (circles), the LLTM fit (solid line), and its 95% CI (band). Statistically significant change in the estimated s2MWT occurs when there is no overlap in 95% CIs between different time points (shown as dashed arrows). The middle panels show the T25FW scores (dots) with 20% thresholds for clinically relevant change (horizontal bars). Similarly, the lower panels show the EDSS scores with its thresholds for clinically relevant change: 1.5 points if EDSS = 0, 1 point if EDSS between 1.0 and 5.5, or 0.5 points if EDSS ⩾ 6.0. Occurrences of a relevant change in clinical outcomes are denoted by asterisks. Abbreviations: T25FW: timed 25-foot walk test; EDSS: Expanded Disability Status Scale; s2MWT: smartphone-based 2-minute walk test; LLTM: local linear trend model.

Using the LLTM, curve fitting was applied on individual patients’ s2MWT scores to derive an estimated trend of ambulatory function over time for each patient. Curve fitting was performed in 87 of the 94 (92.6%) patients as seven patients had less than five s2MWT scores. From the 87 patients’ individual LLTM estimates, the median SD in level (0.013 m) and slope (4.63 × 10^−6^ m) of the fitted trend was much smaller than the median SD of the noise (17.87 m). Thus, a smoothened, less variable trend estimate in ambulatory function was obtained from the highly variable raw s2MWT measurements. Using the algorithm to screen for statistically significant changes, patients’ trajectories in ambulatory function could be categorized as being overall stable (*n* = 48, 55.2%), gradually improving (*n* = 26, 29.9%), gradually deteriorating (*n* = 5, 5.7%), and being overall variable (*n* = 8, 9.2%). For each of these categories, an example of the curve fits together with clinical outcomes is shown in Figure 3. In the visualized patient examples, the 95% CI is generally smaller than the spread in raw measurements, due to the high temporal resolution of s2MWT data.

## Discussion

In this study, an s2MWT was investigated on assessment and monitoring of ambulatory function in MS. The s2MWT had an adherence varying between 45.7% and 65.7%. Reliability was adequate (ICC = 0.76), however, considering a test–retest mean score of 129.5 m, the SDC of 65.6 m was high. Construct validity was found as s2MWT scores were significantly higher in high EDSS patients compared to low EDSS patients and HC, and higher in patients with high compared to low T25FW. Concurrent validity was supported, as the s2MWT correlated moderately to strongly with the T25FW and EDSS. On group level, three monthly changes in s2MWT scores were not responsive to clinically relevant changes in T25FW or EDSS.^13,29^ However, instead of relying on single or a few averaged s2MWT scores, by optimally using the high-frequency smartphone tests, a fitted estimate of ambulatory function on the individual patient level was derived. Thus, improving the temporal resolution and detection of statistically reliable change, which was detected in 39 of 87 (45%) of MS patients.

Adherence to the s2MWT was 65.7% in the initial 4-week period and gradually decreased to 45.7% in M_9_–M_12_. Compared to this, a higher adherence (between 69.4% and 91.5%) was found for a similarly scheduled smartphone cognition test in a previous report.^34^ As the s2MWT is performed outside, it is dependent on weather conditions and less flexible to perform throughout the day than the cognition test. Furthermore, low accuracy of the GPS signal caused some participants to be unable to initiate the walking test, possibly contributing to a lower adherence as well. In a previous cohort of the MS sherpa s2MWT, a 92.4% adherence rate was reported, albeit in a 4-week follow-up in 25 patients with an overall lower age and EDSS than the current study, and with a different definition of adherence.^27^ The FLOODLIGHT 2-minute walking tests found an adherence of 71% (proportion of weeks with ⩾ 3 days of tests) throughout a 24-week follow-up.^35^ The elevateMS 30-second walking test, reported 80.2%–81.1% adherence in the first week that decreased to 46.1%–50.0% in week 12.^26^ Overall, our adherence is slightly lower, but fairly in line with previous reports considering study differences and our adherence rates included all participants with any tests, including drop outs.

When comparing reproducibility and validity parameters, the same s2MWT (MS sherpa) yielded similar estimates and correlation with the EDSS in a previous cohort.^27^ The MSCopilot walking test had higher reproducibility than the s2MWT.^19,25^ This may be attributable to the test being performed under supervision by researchers and being more extensive (20 minutes or 500 m of walking) than our self-assessed s2MWT.^19^ The MSCopilot test was also associated with the EDSS and distinguished patient-groups divided on an EDSS of 3.5, similar to our findings.^25^ A remote unsupervised 2-minute walk test (FLOODLIGHT) was investigated in 76 MS patients and 25 HCs.^20,22–24^ The FLOODLIGHT walking test had reproducibility and concurrent validity with T25FW and EDSS similar to our findings.^24^ Motion sensor data during the walking test demonstrated comparable reliability, but better SDC values than our findings.^22^ This is unsurprising, as there is more variability in walking distance scores calculated on top of sensor data, than sensor data alone. Similar to our findings, the sensor-based features reached moderate to strong correlation with T25FW and EDSS, and distinguished patients with EDSS ⩾ 3.5 from patients with EDSS < 3.5 and HC, whereas the latter two groups were less distinguishable from each other.^20^

Altogether, multiple sensor-based assessments of ambulatory function have been investigated in MS. A strong advantage of the MS sherpa s2MWT is the use of the participants’ own smartphone. The s2MWT can be performed during actual functioning, for example, on the way to the supermarket or during regular walks, and as a smartphone adaptation of the clinically validated 2MWT, is familiar to clinicians and patients. To our knowledge, there are no reports on remote monitoring of ambulatory function by evaluating responsiveness. Our results indicated insufficient group-level responsiveness of the s2MWT to relevant change events in clinical outcomes. This may not be surprising given the high variability in raw s2MWT score, and the T25FW and EDSS being prone to measurement error. Therefore, instead of relying on anchor-based thresholds for change derived from group analyses, a distribution-based method was employed where statistically reliable change is based on individuals’ own scores.

In the distribution-based method utilizing individual-level curve fitting, statistically reliable change was defined to occur when two time points do not overlap in 95% CI bands. While this method is more conservative than computing statistical differences (where variances of two time points are pooled and autocorrelation is subtracted), it is more simple and intuitive. Important to note is that the categorization of curve fitted trajectories in walking function is not (further) analyzed statistically, but used to illustrate how different patient typologies can be distinguished using the estimated s2MWT levels. By allowing monitoring of change visually on the individual level, the method can be easily translated to be used by patients and clinicians. In patients with high temporal resolution s2MWT data, a minimum assessment frequency of once per 11 days was found to still provide an overall robust trend estimate (see Supplementary material). A frequency of once per 7 days better allows consistency and routine. Thus, for practical guidance on individual-based monitoring of ambulatory function, we would recommend weekly measurements and considering non-overlapping 95% CI bandwidths to indicate statistical reliable change.

Our current findings have limitations to be considered. Despite that the follow-up duration of the current study exceeds those in existing reports, it is still short to fully demonstrate the sensitivity in monitoring clinically relevant change in walking function. With relatively short follow-up, real changes in clinical measures tend to be small, whereas the clinical measurements are still affected by measurement variability, and within- and between-rater variability. Another limitation is the missing clinical data due to the COVID-19 pandemic as in-clinic visits were temporarily suspended. This resulted in fewer data for the anchor-based responsiveness analyses. Despite missing clinical visits, however, the distribution-based responsiveness analyses remained largely unaffected as smartphone walking tests were continuously obtained during that period, stressing an advantage of remote digital assessment of function. Finally, naturally occurring measurement variability due to external factors are inevitable. However, a few sources that could potentially introduce unnecessary variability were not entirely accounted for. For instance, the extent to which participants adhered to the walking test instructions was not obtained. In addition, (older) smartphones embedded with lower quality GPS generate lower quality GPS data compared to higher quality GPS smartphones. In the current study, a strict algorithm was therefore used to filter out tests with insufficient GPS quality. For future use or investigation, low-quality GPS can still be used resulting in larger measurement variability (generally wider 95% CI curve fits), which can be partially overcome with higher assessment frequency. Furthermore, the LLTM can be adjusted to include an estimate based on the quality of individual GPS data points in curve fitting.

## Conclusion

The remote, self-performed s2MWT demonstrated clinimetric properties needed for the assessment of ambulatory function. For monitoring ambulatory function, group-level responsiveness to relevant change in clinical outcomes was not demonstrated due to high measurement variability in averaged s2MWT scores and clinical outcomes. By fitting a curve onto all s2MWT scores on the individual patient-level, individual-level responsiveness was demonstrated with the detection of statistically reliable changes in ambulatory function. Further research should aim to investigate the detection of clinically relevant change with this smartphone-based and curve fitting approach.

## Supplemental Material

sj-docx-1-msj-10.1177_13524585231152433 – Supplemental material for Personalized monitoring of ambulatory function with a smartphone 2-minute walk test in multiple sclerosis Click here for additional data file. Supplemental material, sj-docx-1-msj-10.1177_13524585231152433 for Personalized monitoring of ambulatory function with a smartphone 2-minute walk test in multiple sclerosis by Ka-Hoo Lam, Ioan Gabriel Bucur, Pim van Oirschot, Frank de Graaf, Eva Strijbis, Bernard Uitdehaag, Tom Heskes, Joep Killestein and Vincent de Groot in Multiple Sclerosis Journal
