# Supplementary material for: Personalized monitoring of ambulatory function with a smartphone 2-minute walk test in multiple sclerosis
Source: Mult Scler. 2023 Feb 8;29(4-5):606–14. doi: 10.1177/13524585231152433 (PMC10152211; doi:10.1177/13524585231152433)
Supplement: sj-docx-1-msj-10.1177_13524585231152433 – Supplemental material for Personalized monitoring of ambulatory function with a smartphone 2-minute walk test in multiple sclerosis [file sj-docx-1-msj-10.1177_13524585231152433.docx]

**Supplementary material**

**
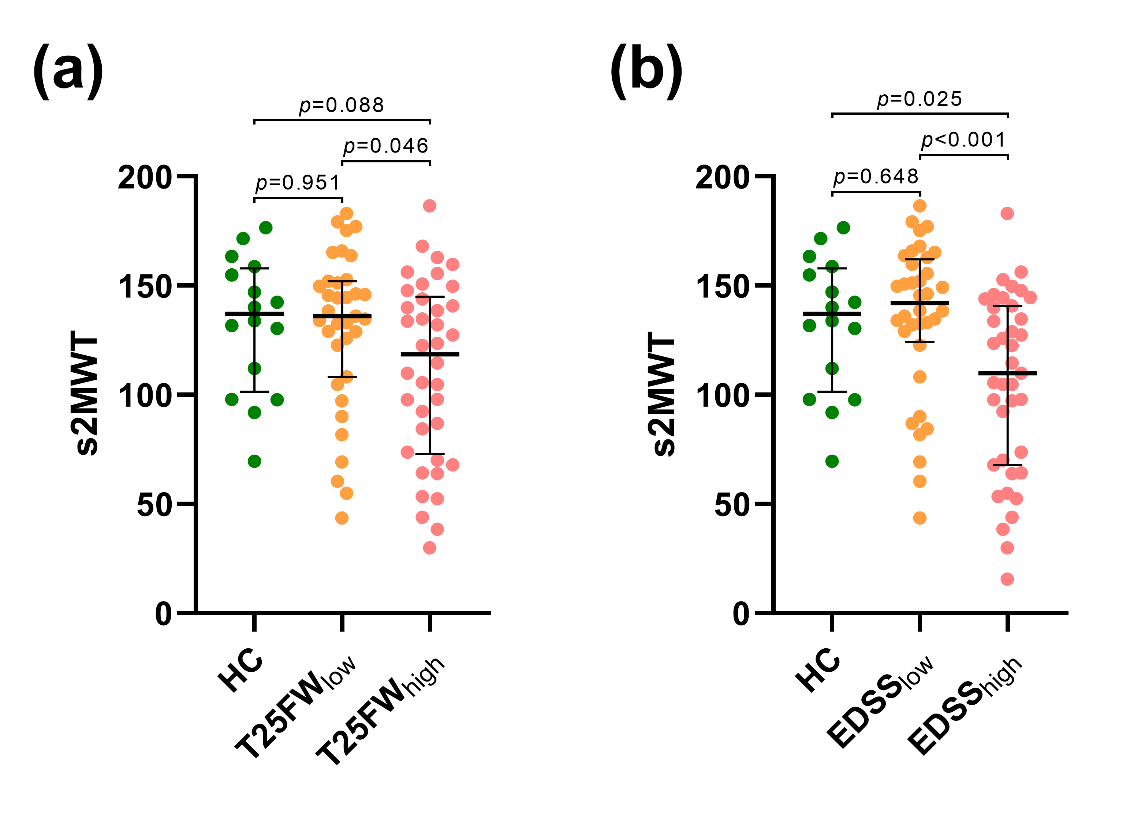
**

**Figure S1.** Scatter dot plots of 2-week averaged s2MWT scores at M_0_ between HC and MS patients split between low and high disability based on: (**a**) T25FW using a median split of 4.7 seconds; and (**b**) EDSS score using a median split of 3.5. Error bars represent the median and interquartile range. *P-*values were based on Mann-Whitney U tests.

Abbreviations: s2MWT, smartphone 2-Minute Walk Test; HC, healthy controls; T25FW, Timed 25-Foot Walk test; EDSS, Expanded Disability Status Scale.

**Table S1.** Spearman correlation coefficients (*ρ*) between clinical and smartphone walking tests in MS patients.

|  | Tests, *n* (median±IQR) | T25FW | |  | EDSS | |  |
| --- | --- | --- | --- | --- | --- | --- | --- |
|  |  | *ρ* | *n* |  | *ρ* | *n* |  |
| s2MWT |  |  |  |  |  |  |  |
| M_0_ | 6.4±3.8 | -0.430*** | 73 |  | -0.443*** | 75 |  |
| M_3_ | 3.3±3.3 | -0.607*** | 57 |  | -0.644*** | 58 |  |
| M_6_ | 2.3±2.4 | -0.507*** | 54 |  | -0.593*** | 53 |  |
| M_9_ | 2.8±3.1 | -0.453* | 31 |  | -0.511** | 33 |  |
| M_12_ | 2.1±2.0 | -0.550*** | 40 |  | -0.524*** | 40 |  |

^ns^not significant, **p*<0.05, ***p*<0.01, ****p*<0.001.

Abbreviations: T25FW, Timed 25-Foot Walk test; EDSS, Expanded Disability Status Scale; s2MWT, smartphone 2-Minute Walk Test.

**The local linear trend model**

The local linear trend model (LLTM) used to estimate the trend in smartphone test scores has been described previously and is restated here.^1^ Natural variability cause day-to-day fluctuations in repeated measurements and are not due to real change in the function that is being measured. Therefore, a better approximation of the true performance of function can be obtained by applying a LLTM (a type of *linear-Gaussian state space model*) on the repeated s2MWT measurements for individual patients.^2^ This de-noises the measurements by taking advantage of high frequency data. The local linear trend fit models the variation in level and slope (that characterize the varying trend, which is interpretable as real change), and irregular residual variation (that characterizes the error or disturbance in measurement).^3,4^ This state space model can be seen as an extension of (maximum likelihood) factor analysis. The measured s2MWT scores can be considered noisy measurements of the underlying latent state factor (i.e., ambulatory function), described as:

$y_{i}^{t}=\mu_{i}^{t}+\varepsilon_{i}^{t}$.

For each patient *i* at time *t*, $y_{i}^{t}$ is the measured s2MWT score, which is equal to the latent s2MWT state ($\mu_{i}^{t}$) plus (zero-mean Gaussian) noise denoted by $\varepsilon_{i}^{t}$ (see Figure S2). The noise is assumed to be stationary, but having a different distribution for each patient. Time is indicated by the discrete index *t*, which is the number of days from inclusion. Days without measurements were treated as missing. The zero-mean Gaussian noise closely relates to measurement error and comprises short-term irregular effects due to factors such as time of the day, mood, level of rest, or the environment. At each time step *t*, the latent s2MWT state is assumed to change according to the following set of equations (the dynamics of the LLTM):

$$\mu_{i}^{t+1}=\mu_{i}^{t}+v_{i}^{t}+\xi_{i}^{t}$$

$$v_{i}^{t+1}=v_{i}^{t}+\zeta_{i}^{t}$$

The latent state consists of its *level* $\mu_{i}^{t}$ and *slope* $v_{i}^{t}$. The level term models how the latent s2MWT state changes over time, while the slope term models its rate of change. The change in state level at each time point is given by the current slope plus random patient-specific zero-mean Gaussian noise $\xi_{i}^{t}$. The slope follows a random walk with patient-specific zero-mean Gaussian noise $\zeta_{i}^{t}$. Thus, fitting this model to the data of a specific patient involves estimating three parameters: the irregular (residual) variance (Var[$\varepsilon_{i}^{t}$]), the level variance (Var[$\xi_{i}^{t}$]), and the slope variance (Var[$\zeta_{i}^{t}$]). Concurrently, the best-fitting (maximum likelihood) latent state value is estimated at each time point. These results are obtained with maximum likelihood estimation, as implemented in the Python package *statsmodels.*


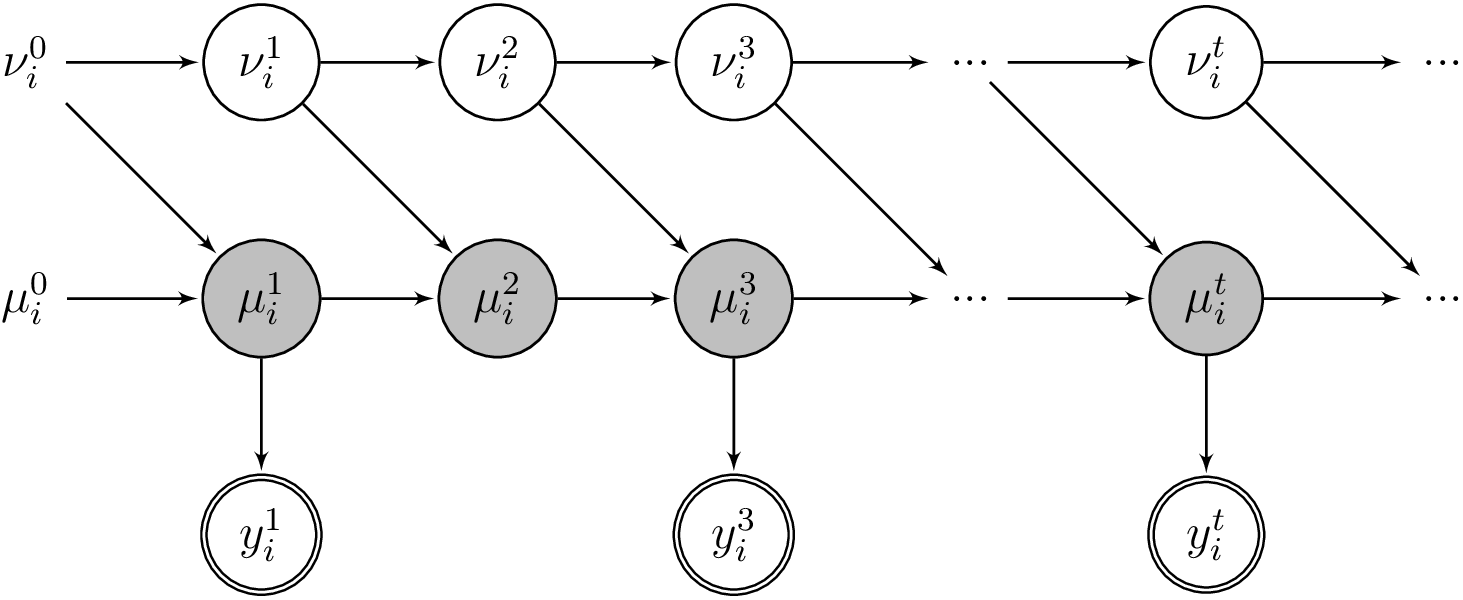


**Figure S2.** Diagram of local linear trend model.

The local linear trend model consists of two hidden state variables ($\mu$, the level, and $\nu$, the slope) that evolves over time. The latent s2MWT scores (levels) are highlighted in grey, and are measured with error $\varepsilon$. The observed or measured variables are marked with a double circle. Note that the model naturally handles missing values, as it does not require measurements for each time step.

Abbreviations: s2MWT, smartphone 2-Minute Walk Test.

**Categorization of s2MWT trajectories**

An algorithm was developed to screen for statistically significant changes using the estimated s2MWT trend. Essentially, the time series were scanned in an efficient manner for two days that show no overlap between the 95% CI bands. Each confidence band has a lower and upper bound, which is denoted (for patient *i* at time *t*) by $l_{i}^{t}$ and $u_{i}^{t}$, respectively. The algorithm works by keeping track of two quantities: $u^{min}$, the smallest upper bound value found (so far) in the scan; and $l^{max}$, the largest lower bound value found (so far) in the scan.

The scan begins by initializing the above quantities using the baseline estimated confidence band: $u^{min}=u_{i}^{0}, l^{max}=l_{i}^{0}$, where $u_{i}^{0}$ and $l_{i}^{0}$ are the lower and upper bounds of the 95% CI band at baseline (day zero). At each step, the upper and lower bound of the CI band of subsequent days is compared to these quantities, and updated if necessary ($u^{min}$ is updated if a smaller upper bound value is found, $l^{max}$is updated if a larger lower bound value is found). The scan is stopped when a significant difference is found, that is either $u_{i}^{t}\leq l^{max}$or $u^{min}\leq{l_{i}}^{t}$. In the former case, the 95% CI upper bound of the estimated trend at the current day is lower than the highest lower bound value found in the scan. Since the upper bound of the current day is lower than the lower bound of a previous day, a significant negative change has occurred (the bands do not overlap). Analogously, in the latter case, a significant positive change has occurred because the lower bound of the current day has a higher value compared to the upper bound of a previous day in the scan. Although a significant change has been found, the scan is continued to find the largest significant change, that is, if the estimated trend continues to increase (or decrease) when a significant improvement (or deterioration) has been found. Once the largest significant change has been found, the current day (in the scan) is chosen as the new starting point, and the scan is continued for the next significant change. At the end of the scan, all statistically significant changes (positive or negative) that have been found are collected, and that information is used to categorize patients into four distinct categories:

1. *Stable* – if no significant changes were found;
2. *Improvement* – if only significant positive changes were found;
3. *Deterioration* – if only significant negative changes were found;
4. *Variable* – if both positive and negative significant changes were found.

**Assessment frequency**

To investigate the impact of s2MWT assessment frequency on the 95% CI width (i.e., the distance in meters between the upper and lower bound), six patients with the highest frequency of performed s2MWTs were selected. These six patients performed s2MWTs with at most a week between tests, for at least 72 successive measurements, which allowed downsampling of their s2MWT assessment frequency for the purposes of this investigation. Starting from the first data point, we considered the first *N* data points for each patient, where *N* is determined by the patient with the shortest follow-up period. For these patients, the change in CI bandwidth was then assessed for different assessment frequencies by downsampling the number of s2MWT assessments. For the downsampling, every *n*-th data point was taken in the remaining dataset of *N* data points per patient. The *n* was varied between 1 (all *N* data points from the selected patient) and 15 (selecting every 15th data point from the selected patient).

For each obtained assessment frequency, we derived a (simplified) average measure of statistically significant change, in a similar vein to how the SDC is derived in clinical practice. For each patient, we considered the standard deviation of the estimated s2MWT latent level at each of the *N* data points. We then multiplied these values by $1.96\times\sqrt{2}$ to obtain an SDC equivalent, which we here call the ‘change bandwidth’ (and is equal to the 95% CI bandwidth in Figure 3, divided by $\sqrt{2}$). We averaged the values obtained for all data points per patient, and we repeated this calculation for each of the six patients. In Figure S3 the ‘change bandwidth’, expressed as a percentage of the average estimated s2MWT distance walked at baseline across 87 patients, is visualized as a function of varying s2MWT assessment frequency. The estimated s2MWT distance walked, averaged over 87 patients at M_0_ was 126.3 m (SD=42.8 meters). If a 20% (25 meters) MCID^5^ in the s2MWT score is considered, at least one measurement every 11 days is needed to detect clinically relevant change with sufficient confidence (see also Figure S3).


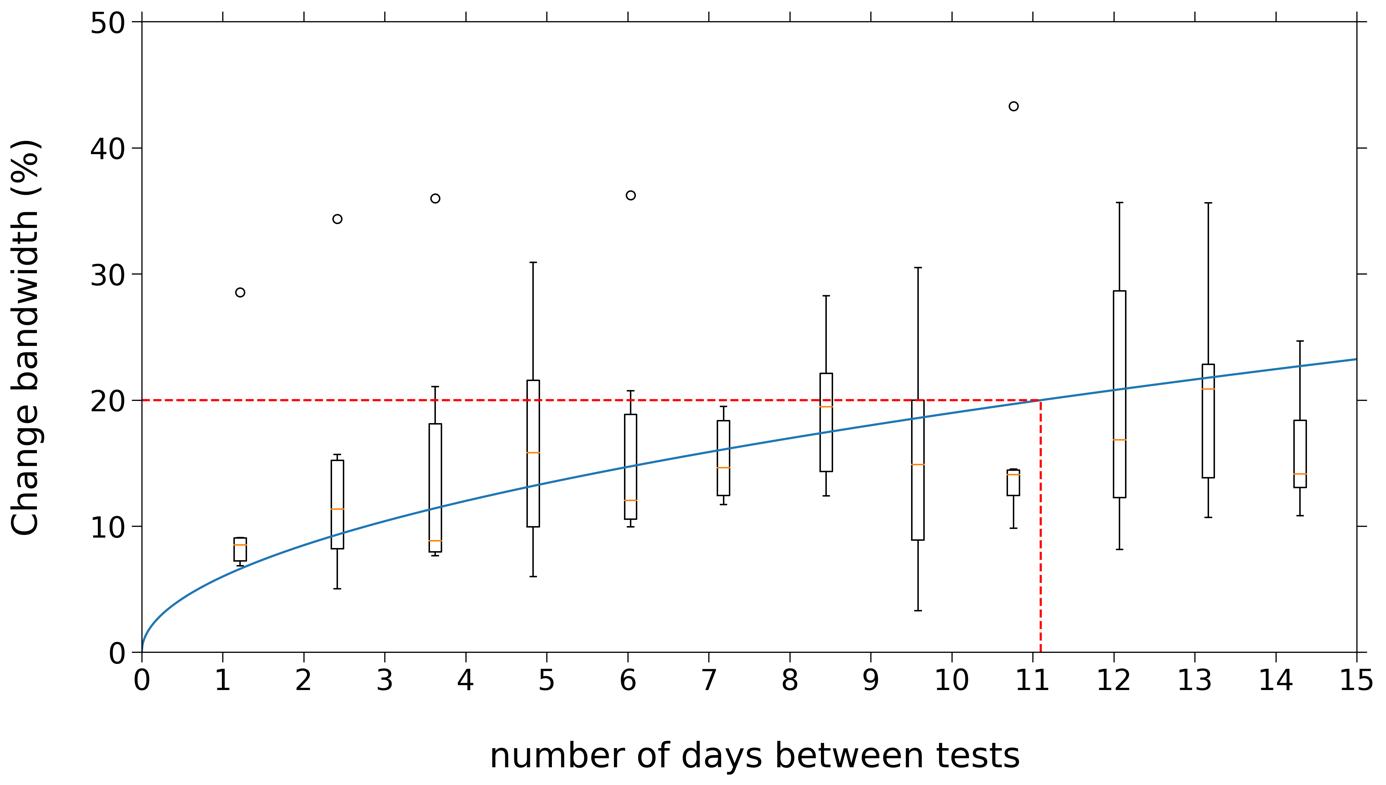


**Figure S3.** Box plots visualizing the ‘change bandwidth’ in six selected patients with the highest s2MWT assessment frequency, as a function of (downsampled) assessment frequency. The superimposed blue curve shows how the relationship between the change bandwidth and the number of days between tests roughly follows a ‘square-root-of-*N*’ law, *N* being the size of the data, as we would expect for the standard error.^6^

**References**

1. Lam KH, Bucur I, Van Oirschot P, De Graaf F, Weda H, Strijbis E, et al. Towards individualized monitoring of cognition in multiple sclerosis in the digital era: A one-year cohort study. Multiple sclerosis and related disorders. 2022;60:103692.

2. Durbin J, Koopman SJ. Time Series Analysis by State Space Methods: Second Edition: OUP Oxford; 2012 2012/05/03/. 369 p.

3. Oirschot Pv, Heerings M, Wendrich K, Teuling Bd, Martens MB, Jongen PJ. Symbol Digit Modalities Test Variant in a Smartphone App for Persons With Multiple Sclerosis: Validation Study. JMIR mHealth and uHealth. 2020;8(10):e18160.

4. Pham L, Harris T, Varosanec M, Morgan V, Kosa P, Bielekova B. Smartphone-based symbol-digit modalities test reliably captures brain damage in multiple sclerosis. NPJ digital medicine. 2021;4(1):36.

5. Hobart J, Blight AR, Goodman A, Lynn F, Putzki N. Timed 25-foot walk: direct evidence that improving 20% or greater is clinically meaningful in MS. Neurology. 2013;80(16):1509-17.

6. Altman DG, Bland JM. Standard deviations and standard errors. BMJ (Clinical research ed). 2005;331(7521):903.
